# From the Age of 5 Humans Decide Economically, Whereas Crows Exhibit Individual Preferences

**DOI:** 10.1038/s41598-017-16984-0

**Published:** 2017-12-06

**Authors:** Samara Danel, François Osiurak, Auguste Marie Philippa von Bayern

**Affiliations:** 1 Laboratory for the Study of Cognitive Mechanisms, University of Lyon, Rhône-Alpes, Bron, 69676 France; 2 University Institute of France, Paris Ile-de-France, Paris, 75005 France; 3 Max-Planck-Institute for Ornithology, 82319 Seewiesen, Germany; 4 Department of Zoology, University of Oxford, Oxford, OX1 3PS UK

## Abstract

Human societies greatly depend on tools, which spare us considerable time and effort. Humans might have evolved a bias to employ tools, using them even when they are unnecessary. This study aimed to investigate whether adult humans and a distantly related habitually tool-using vertebrate species, the New Caledonian crow (*Corvus moneduloides*), use tools depending on their necessity. In addition, children aged 3 to 5 years were examined to investigate the developmental pattern. The task involved choosing between using a body part (i.e. crows: beak; humans: hand) or a tool for retrieving a reward from a box. All subjects were tested in two conditions. In the Body+/Tool− condition, using the body was more efficient than using the tool, and conversely in the Body−/Tool+ condition. Our results suggest that the capacity to employ tools economically develops late in humans. Crows, however, failed to choose economically. At the individual level, some subjects exhibited striking individual preferences for either using a tool or their beak throughout the task. Whether such biases depend on individual experience or whether they are genetically determined remains to be investigated. Our findings provide new insights about tool use and its cognitive implementation in two outstanding tool-using taxa.

## Introduction

How could humans do without tools? From the small hunter-gatherer communities to the bewilderingly complex industrialized western societies, tools are found everywhere. The assumed link between tool use and the evolution of the human brain has spurred research into the cognitive abilities associated with tool use^1–4^.

For instance, studies involving patients with neuropsychological syndromes (e.g. apraxia: a disorder of learned gestures, in the absence of sensory or motor deficits^5^) have significantly contributed to a better understanding of human tool-related cognition^6–8^. We know now that some human tool-use activities involve a diverse set of cognitive capacities, such as semantic reasoning, working memory, simulation-based decision-making, or technical reasoning^9–11^.

Over the last decades, research in experimental psychology has suggested that tools also implicitly affect the way we perceive the world^12^. These studies are based on earlier findings that human spatial perception is influenced by locomotor effort^13,14^, and report that a similar pattern is found in a tool-related context. For example, adult humans perceive a distant target closer when they intend to reach it with a tool rather than with the hand, and this also occurs when the subject passively observes a tool-use action made by another individual^15,16^. The emergence of this underestimation is most probably tightly linked to the prepotent role of tool use throughout human evolution^17,18^.

A similar finding is observed when humans have to decide when to use a tool. Tool use is not always more efficient than using one’s hands, but our dependency on tools as well as their abundant availability might have progressively biased the way we perceive the benefits, but also the costs associated with tool use. A recent study explored this question, by investigating how adult humans estimate the costs and the benefits of tools in motor tasks^19^. Subjects had to move different quantities of objects by hand (2 at a time) or with a tool (4 at a time). The tool was out-of-reach, so the participants had to fetch it first before moving the objects. Interestingly, humans tended to perceive tool actions as less costly than they actually were. More importantly, when using the hand was slightly more beneficial than using the tool, subjects preferred to use a tool to solve the task. These results suggest that adult humans do not always behave economically when using a tool. Rather, they seem to have developed a tendency to overestimate the benefits conferred by tool use probably through social and asocial learning, but possibly also including some genetic factors. This propensity, however, gets masked the more conspicuous the difference in terms of time and effort between using the tool and using the hand becomes^19^. In this case, subjects rather tend to minimize the effort required for accomplishing the task, a phenomenon known as the principle of least-effort^20^.

Whether children have similar biases and how differently aged children perceive the costs/benefits of tools has not been investigated. Although, by the age of 2, children are proficient tool users^11^ there are still few studies on tool use in children and the developmental trajectories remain poorly investigated^21–27^. It is known that from at least 18 months children start manipulating and using a wide range of tools, and that they learn how tools work by watching others^28^. However, whether they understand the benefits a functional tool confers or whether other motivations drive their interactions with tools remains unexplored. So far, the only related experiment in the field confronted preschool and primary school children with a task where they could choose between getting a reward either by pushing a lever, or by grasping it directly from a container^29^. In this study, 4-year-olds chose to push the lever significantly more than to use their hands to get the reward. Pushing a lever does not meet the criteria of tool use strictly speaking^30^. However, it serves as indirect evidence that by the age of 4, children may either lack the ability to estimate the benefits provided by tool use accurately or they might be biased to interact with tools.

Similarly, it is not known how tool-using animals judge the pay-offs of tool use accurately, or what other processes may drive their tool use. The New Caledonian crow, a species belonging to the large-brained and cognitively very advanced corvid family^31^, is one of the two avian species known to use stick tools habitually to extract woodboring beetle larvae in the wild (together with the Galapagos woodpecker finch *Cactospiza pallida*)^32,33^. It is also the only avian species that manufactures and uses several types of tools (from non-hooked stick-type tools e.g., bamboo stems, tree twigs or fern stolons, to various kind of hooked tools e.g., sticks or stepped-cut *Pandanus* spp. leaves^34,35^) for different purposes, such as food extraction and information gathering^36^. This includes the arguably most complex tool manufacture technique described in animals, which potentially may have arisen through cumulative cultural processes^37^, although the latter remains debated^38^. This species therefore represents a relevant biological model for investigating the factors behind technological evolution in hominins^39^. Other than New Caledonian crows, only humans and a few non-human primate species have shown diversity in their tool types^40,41^. Given these functional similarities, it is of particular interest to find out how proficient New Caledonian crows are at judging the costs/benefits afforded by tool use, and whether we share similar psychological tendencies biasing them into using tools frequently.

The goal of this study was therefore to investigate how (un)economic decision making in the context of tool use develops in humans, and, second, how it is expressed in a phylogenetically distant tool-using taxon.

**Figure 1:**
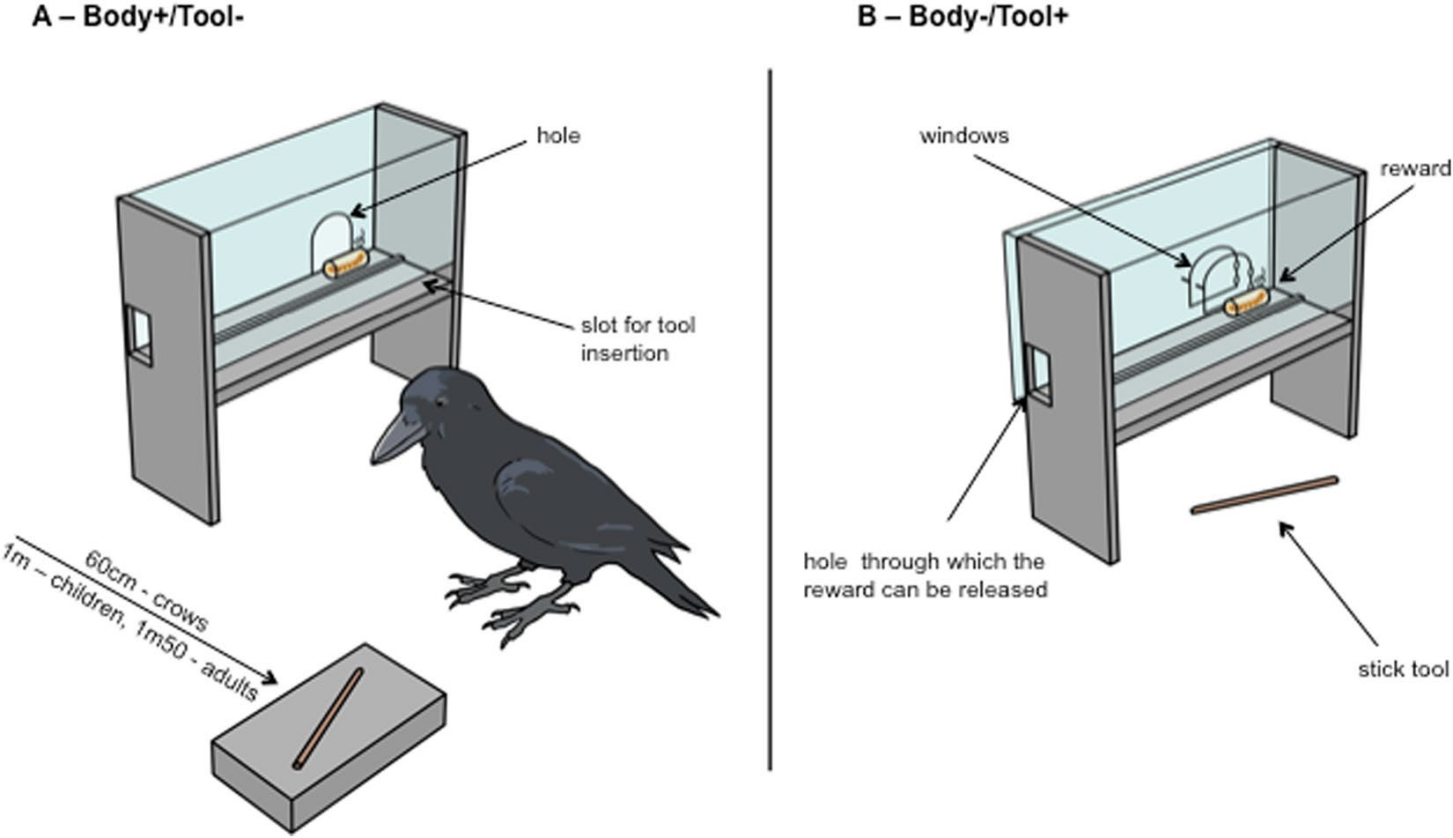
Schematic representation of the test boxes used in the two conditions in New Caledonian crows, 3- to 5-year-olds and adult humans. Drawing courtesy of Nicolas Brachet. (**A**) Body+/Tool− condition, where using the hand/beak was quicker than using the tool. The subjects had to choose between either directly taking the reward with their hand/beak out of the hole in the box, or fetching the stick in order to slide the reward out along the track towards the little side on the left. The stick was presented on a wooden platform, and was placed at a distance from the box (crows: 60 cm, children: 1 m, adult humans: 1m50). (**B**) Body−/Tool+ condition, where using the tool was quicker than using the hand/beak. The subjects had to choose between either opening two windows successively in order to take the reward with their hand/beak, or using the stick located just in front of the box to slide the reward out of the box.

Fifteen adult humans, 19 3-year olds, 9 4-year-olds and 22 5-year-olds as well as 8 New Caledonian crows participated in this study. All subjects were confronted with 2 opposite conditions in which they had to choose between either using a body part (i.e. beak: crows; hand: humans) or a tool to access a reward in a box. In the Body−/Tool+ condition, using the tool was more efficient than using the body. In the Body+/Tool− condition, however, using the body was more efficient than using the tool (Fig. 1). In this context, we define behaving economically (in terms of time and effort) as using the tool in the Body−/Tool+ condition and the body in the Body+/Tool− condition. The crows were also confronted with additional tests (i.e. a transfer task and a novel object exploration task) to assess whether potential individual behavioural preferences for using the beak or the tool persisted in other contexts. This experiment is the first at aiming to evaluate the estimation of costs/benefits associated with tools in adult humans, human children and a non-hominid tool-user.

## Results

### Humans

**Figure 2:**
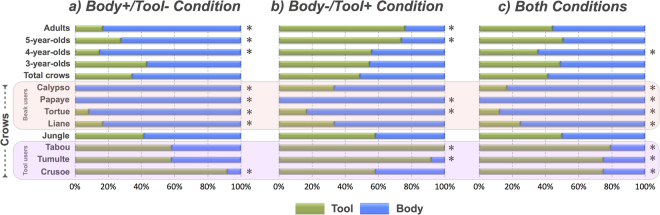
Distribution of the choices made for the tool versus the hand/beak by adult humans, children of the 3 different age groups and the crows during the test phase. The performance of each group is represented for the Body+/Tool− condition, the Body-/Tool+ condition, and both conditions respectively. For the crows, individual preferences for either the tool or the beak in each condition in the test phase are reported additionally. Labels ‘beak users’ and ‘tool users’ represent the subjects that have shown a significant preference for using the beak or the tool throughout the test phase, respectively. Wilcoxon and binomial tests were used to assess preference at the group and the individual level. Asterisks (*) denote significance (p < 0.05).

Adult humans used tools significantly more than their hand when they provided an advantage in the Body−/Tool+ condition (Wilcoxon paired *T*-test: *Z* = 2.34, *p* < 0.02), and conversely less in the Body+/Tool− condition (Wilcoxon paired *T*-test: *Z* = 2.70, *p* < 0.01; Fig. 2). This pattern resulted in no preference when both conditions were gathered together (Wilcoxon paired *T*-test: *Z* = 0.44, *p* > 0.05). The same held true for 5-year-olds (Wilcoxon paired *T*-tests: Body−/Tool+: *Z* = 3.91, *p* < 0.001; Body+/Tool−: *Z* = 3.47, *p* < 0.001; Both: *Z* = 0.12, *p* > 0.05; Fig. 2). However, no significant difference was found in 4-year-olds in the Body−/Tool+ condition (Wilcoxon paired *T*-tests: *Z* = 1.4, *p* > 0.05; Body+/Tool−: *Z* = 2.80, *p* < 0.01; Both: *Z* = 2.24, *p* < 0.01; Fig. 2), and in 3-year-olds in neither condition (Wilcoxon paired *T*-tests: Body−/Tool+: *Z* = 1, *p* > 0.05; Body+/Tool−: *Z* = 1.11, *p* > 0.05; Both: *Z* = 0.17, *p* > 0.05; Fig. 2).

### New Caledonian crows

Subjects chose indifferently at the group level, so no significant difference between the two options was detectable in either condition (Wilcoxon paired *T*-test: Body−/Tool+: *Z* = 0.21, *p* > 0.05; Body+/Tool−: *Z* = 1.19, *p* > 0.05; Both: *Z* < 1.19, *p* > 0.05; Fig. 2).

### Distribution of individual differences in crows and humans

**Figure 3:**
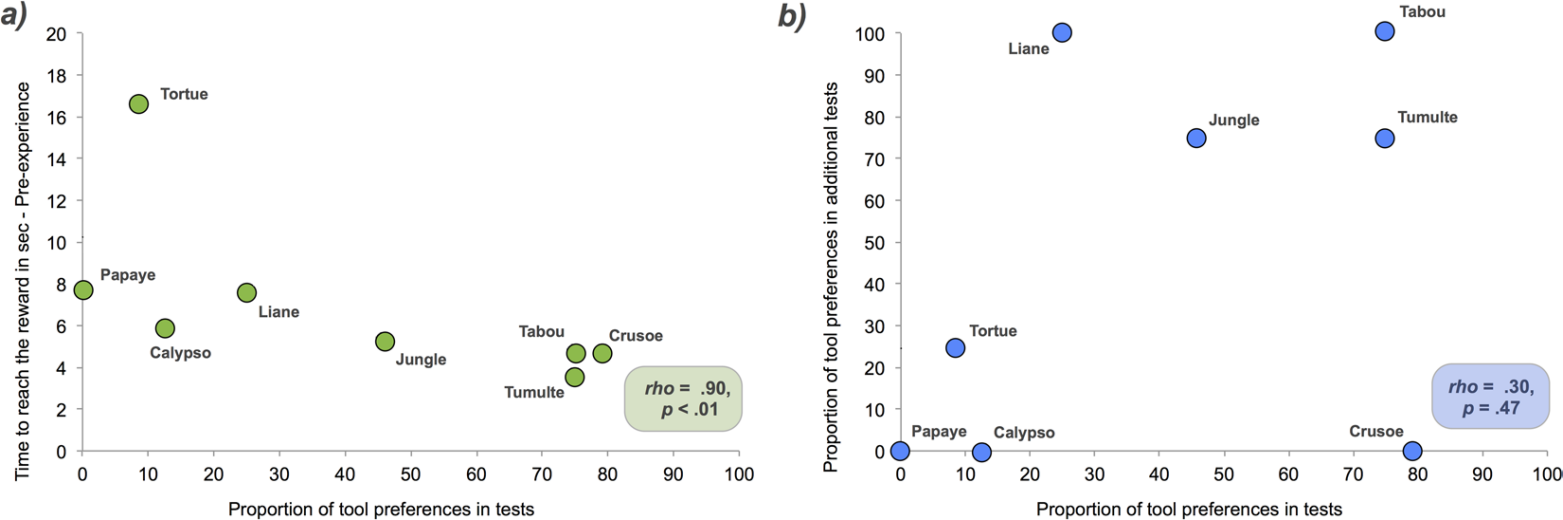
Representation of the crows’ individual tool preferences in the test phase, in relation to the pre-experience phase and the additional tests. (**a**) Relation, in seconds, between the time spent to reach the reward with the tool in the pre-experience phase, and the tool preferences in the test phase. (**b**) Relation between the proportion of tool preferences of the total trials for the test phase and for the additional tests. In the test phase, the proportion of the total 24 trials in which the tool was preferred over the beak were collected (total of the Body+/Tool− condition and the Body-/Tool+ condition, maximum = 12 tests in each condition). The proportion of tool preferences in the additional tests was calculated from the 3 trials of the transfer task and the single trial of the novel object exploration task.

Individual performance (binomial probabilities reported in Fig. 2) indicated a substantial variation between crows, which was particularly apparent when optimal decision-making in both conditions was considered together. Regardless of the conditions in the test phase, 4 subjects significantly preferred using their beak (i.e., the so-called ‘beak users’: Liane, Papaye, Tortue and Calypso), 3 significantly preferred using the tool (i.e., the so-called ‘tool users’: Tabou, Tumulte, Crusoe), whereas 1 (Jungle) showed no preference. Besides, a significant negative correlation between the proportion of tool preferences in the test phase and the time to reach the reward with the tool in the pre-experience phase was obtained (*rho* = 0.90, *p* < 0.01; Fig. 3a). Further analyses were conducted in order to examine whether the individual differences observed in crows would also be found in humans. Binomial probabilities were first computed for each subject of each group individually in both conditions (Body+/Tool− and Body−/Tool+). This analysis allowed us to determine whether a subject showed a preference for the Tool, the Body, or no preference. The numbers of subjects with each type of preference for each group are reported in Table 1. Most crows (88% of subjects) showed a preference for either the Tool or the Body while most adults and 3- to 5-year-olds had no preference (78% of subjects). We performed *χ* ^2^ analyses, which confirmed this pattern of results (*χ* ^2^ = 26.21, *df* = 8, *p* < 0.01). The cells that most contributed to the *χ* ^2^-value were the 3 cells occupied by crows (partial *χ* ^2^-value based on these 3 cells: 13.67, that is, 52% of the *χ* ^2^-value; note that for *df* = 8, a *χ* ^2^-value of 15.51 is significant at *p* < 0.05).

**Table 1:** Distribution of individual differences in adults, children and crows in the test phase (both conditions).

	Preference		
	Tool	Neither	Body
Adults	***20***	***53***	***27***
	(*n* = 3)	(*n* = 8)	(*n* = 4)
5-year-olds	***0***	***100***	***0***
	(*n* = 0)	(*n* = 22)	(*n* = 0)
4-year-olds	***0***	***78***	***22***
	(*n* = 0)	(*n* = 7)	(*n* = 2)
3-year-olds	***10***	***74***	***16***
	(*n* = 2)	(*n* = 14)	(*n* = 3)
Crows	***38***	***12***	***50***
	(*n* = 3)	(*n* = 1)	(*n* = 4)

Numbers in bold and italics refer to percentages computed for each group separately. Numbers in brackets are numbers of individuals concerned. Each cell represents the percentage/number of subjects showing a significant preference for either the tool, the body, or neither based on individual binomial tests in both conditions (Body+/Tool− and Body-/Tool+).

### Additional tests for the crows

We checked if the individual preferences for using the beak or a tool persisted in additional tests (see Supplementary Information). Four data points were collected: the performance in the 3 trials of the transfer task and the performance in the single trial of the novel object exploration task. Four out of the 7 crows that showed a significant preference towards one option in the test phase (Tool users: Tumulte and Tabou; Beak users: Papaye and Calypso) persisted in using this option in additional tests, so only a weak pattern was discernible. We computed Spearman rank order correlations appropriate for small samples. No significant correlation between the proportion of tool preferences in the test phase and those in the additional tests was found (*rho* = 0.30, *p* = 0.47; Fig. 3b).

## Discussion

Whereas adult humans may exhibit a tendency to overestimate the benefits conferred by tools^19^, the cost/benefit difference in terms of time and effort seemed to have been clearly perceivable for adult humans in our task. They accurately weighed up between the two options and decided for the less costly/more beneficial one irrespective of whether it involved tool use or use of their hand (Fig. 2). The pattern was mirrored by the 5-year-olds. Like the adults, they appeared to perceive the differences in costs/benefits between the two options and decided accurately for the most economic option, using hands or tools flexibly depending on what was less costly (Fig. 2). They also seemed aware of the functionality the tools conferred, and employed them in a goal-directed manner^42,43^.

However, a strong developmental effect became apparent in the younger age groups (Fig. 2). The 3- to 4-year-olds did not behave economically nor did they show any bias for using tools, irrespective of whether they provided a benefit or not. In fact, most chose randomly by showing no preference for using either the tool or the hand, and irrespective of the condition (Table 1).

A pre-requisite for understanding the developmental effect in this study, i.e., the failure of the younger children to employ tools optimally, is a more precise picture of what cognitive abilities underpin the economic decision making of the adults and possibly also the 5-year-olds in the first place. How do they weigh up between the two options? Do they mentally represent the time and effort associated with both possible actions?

One possibility is that younger children may lack the cognitive skills necessary for judging the cost of different motor actions in terms of time and/or effort properly in order to make economic decisions. Alternatively, younger children may struggle with the task because the cost difference in terms of time and effort between the two options is still not sufficiently pronounced for them to perceive it. For example, it is possible that younger children’ perception of time might still be very crude, so that they may only differentiate between extremely high-contrast durations. It seems unlikely, however, that time perception is the factor constraining the performance of the 3- to 4-year-olds. There is experimental evidence that children can judge time from an early age^44–47^ (although this ability can be considerably constrained by limits in their span of attention, thus the latter could be a confounding factor)^48^. The sensitivity to duration, however, increases with age^49^, which may explain why the ability to decide economically whether to use a tool seems to emerge only at the age of 5. A better understanding of the representation of temporal events in children and its ontogeny is required before this question can be resolved.

On the other hand, the ability to perceive effort precisely is influenced by age and cognitive development in humans^50^. Interestingly, this capacity is emerging at the age of 5^51^. This suggests that effort perception may represent a constraining factor in 3- to 4-year-olds preventing them from being able to choose economically, and it could be explained by the lack of physical experience of this age group^52^. Consequently, time and effort perception clearly could have been sufficient guiding principles in the decision making process in both adult humans and 5-year-olds.

Another dimension that needs to be considered regards the social nature of the task. It is a possibility that the difference seen between 5-year-olds and younger age groups is explained by the more developed social skills of the older children and their capacity to represent others’ intentions^53^. Particularly given that the testing took place within the usual formal educational setting and in the presence of an experimenter, the older children may have tried to understand the experimenter’s goal so as to meet her expectations, acting economically as a result. In contrast, younger children would not be sensitive to the ‘intended’ context of the task and might have failed to behave economically for this reason rather than because of a lack of other skills. In order to verify this, the experiment would have to be repeated without the presence of an adult experimenter, which is practically difficult when testing children of this age range.

Evidence from adult humans suggests that they are capable of anticipating the effort associated with their actions^12^, but without necessarily construing an objective representation of the physical world. For instance, people who are physically fatigued, of poor health or encumbered (e.g., wearing a heavy backpack), overestimate distances and the slants of hills^13,14^. Interestingly in this respect, people may also perceive targets as to be reached easier when using or anticipating the use of a tool rather than the hand^16^.

However, as discussed above, the overestimation of the benefits provided by tools as reported by Osiurak *et al*. (2014) did not occur in adults, nor did we find it in 5-year-olds, maybe because the cost difference between the two options was sufficiently pronounced. In Osiurak *et al*.’s (2014) study, the conditions appeared less contrasted in terms of time and effort, making it more difficult to perceive that one option was more costly than the other. This might have revealed an existing bias for using tools, which would not have become apparent in more contrasted situations, such as the present study. Although this more pronounced difference in costs in our study may have been less obvious for the younger age groups, they also clearly seemed to have no bias for tools.

Contrary to the humans, the crows did not show any preference towards the use of either the beak or the tool, irrespective of the condition at the group level, nor did they appear to behave economically at the individual level (as discussed below; Fig. 2). The crows’ failure could be explained in different ways.

First, they might lack the cognitive skills necessary to make economic decisions in the context of tool use. Deciding which of the two options is more efficient requires assessing the amount of time and effort linked to each of them and weighing up between them. Second, the task also requires some sort of inhibitory control or at the least motor self-control, given that the food and the tool might constitute salient stimuli that the animals may respond to impulsively. It is possible that the crows might not exhibit these skills. However, this seems unlikely considering that New Caledonian crows have been shown to possess remarkable cognitive skills allowing them e.g. to plan an action sequence involving several steps in order to reach a particular goal^39^, which arguably also involves reasoning about the most efficient route to solve a given problem in terms of time and effort. They are also good at inhibiting impulsive motor actions^54^ and they should be able to master the temporal dimension of our task as well, given that corvids of the same genus have been shown to be capable of weighing up between different options at different points in time^55^.

Another interesting pattern other than economic decision-making became apparent when examining individual performance. Seven out of 8 subjects showed a significant preference towards one option (i.e. the beak users and the tool users) in the test phase, coupled with a substantial inter-individual variability. Interestingly, the individual preference persisted throughout the additional tests for some subjects, suggesting potential personality differences^56^ (Fig. 3b). A first possibility to explain these individual differences is the degree of shyness or neophobia. Though New Caledonian crows encounter few predators in their natural environment^34,40^ they may still show neophobic responses and often use tools if they are scared to touch a novel object directly with their beak^36^. This could bias shyer individuals into using tools even if they convey no economic benefit. Or it could affect older, wild-caught individuals which might be scared by the apparatus, but this was not supported by our results. Three wild-caught subjects actually preferred to use the beak in both conditions of the test phase, and completed the pre-test phase with nearly no neophobic behavior.

Another possibility is that there are individual differences in the propensity to use tools within the population, with some individuals using tools more readily than others. Individual differences in behaviour within a species is common^57,58^. Even if tool use and tool manufacture in New Caledonian crows have been proposed to be species-wide^59^, i.e. most populations use tools regularly in the wild, the degree of which each individual does it remains unknown. The reason behind those individual differences remains to be investigated. It may be explained by the individual histories of experience with tools. Individuals with more exposure to tools might have developed improved motor skills and therefore prefer tools. They might have become so efficient in their tool use that using a tool would take them only slightly longer than using their own beak in situations where they could reach food without a tool. Consequently, they would not *perceive* the use of the tool as costly. However, though we found that, in the pre-experience phase of the Body+/Tool− condition, the crows which preferentially and persistently used the tool throughout the test phase and the additional tests (Tabou and Tumulte) seemed more dexterous, spending less time to get the reward than those which used preferentially their beak (Tortue and Papaye) (Fig. 3a), these competent tool users still took longer to reach the reward with the tool rather than with their beak. This further suggests that the crows did not seem to decide economically. While this sample is too small to make any firm conclusions, it highlights the significance of studying the development of individual differences within the same species^60^. These latter may originate from multiple factors, ecological, genetic and/or developmental, which might play an important role in determining each individual tool-related history.

In the wild, New Caledonian crows (juveniles as well as adults) invest considerable time and energy when foraging with stick-type tools for larvae. Generally, much practise as well as social and asocial learning^40^ is necessary to develop the fine sensorimotor skills and motor control, which make this foraging technique profitable^34^. Though this behaviour is costly, it is subsequently compensated by the high nutritional value^61^ of the prey. In this regard, we need to be cautious when comparing cognitive mechanisms among such distant species. The same task might be perceived very differently by crows and humans and would thus not measure the same, which is a common drawback faced by many comparative studies^62^.

In conclusion, our results show that 5-year-olds and adult humans seem able to decide economically in an optional tool use context, i.e. they employed tools depending on their efficiency, while younger children and crows did not.

The crows behaved economically neither at group nor at individual level. Instead, most exhibited striking individual preferences for either the tool or the beak option irrespective of which option was more efficient, and not all of the crows persisted in using their preferred option during additional tests. Further study is necessary to determine the existence of such variation in the wild and to understand to what extent it might be genetically determined. Also, it needs to be studied how such preferences develop in individual crows, by controlling and varying the subjects’ tool related experience experimentally. Further follow-up studies are required to investigate whether the tendency of adult humans and 5-year-olds to behave economically persists if they are tested in additional tests comparable to those of the crows. Additionally, further experiments on younger children are necessary to determine how this skill develop, and whether young children would show economical behavior if they are given more experience in the pre-experience phase.

Finally, this study did not aim at finding an all-or-nothing phenomenon, nor to describe a typically human characteristic. The ability to decide economically could represent an adaptive capacity among humans, and we need to consider it according to the function that it serves for each species. Indeed, considering crows’ morphological and behavioral features^31^, why should they behave economically? These birds live in an environment with few predators and spent a substantial amount of time foraging in their environment as well as interacting with tools. Given their high disposition and motivation to interact with tools in the wild, their failure to respond to this little pronounced difference in costs in terms of time and effort in this very specific task, may not fully reflect their cognitive capacity for economic decision making capacity. This highlights the need of further studies with higher ecological validity and a sharper contrast in costs and benefits between tool and beak use.

## Methods

### Subjects

#### Humans

Fifteen healthy adult subjects with normal or corrected-to-normal vision took part in this study (6 males and 9 females, 100% Caucasians, mean age = 33 years; SD = 9.91 years). Fifty healthy children between 3- to 5-year-olds with normal or corrected-to-normal vision were recruited in this study (96% Caucasian, 2% North African, 2% South African). Children were divided into 3 age groups: 3-year-olds (N = 19, 9 males and 10 females, mean age = 3 years: 7 months, range = 3 years: 1 month - 3 years: 10 months, SD = 2.55 months), 4-year-olds (N = 9, 4 males and 5 females, mean age = 4 years: 10.5 months, range = 4 years - 4 years: 11 months, SD = 0.75 months) and 5-year-olds (N = 22, 7 males and 15 females, mean age = 5 years: 4 months, range = 5 years: 1 month - 5 years: 7 months, SD = 2.23 months).

#### Crows

Eight New Caledonian crows (*Corvus moneduloides*) participated in this experiment (5 females: Liane, Tortue, Tabou, Tumulte and Calypso and 3 males: Jungle, Papaye and Crusoe). Except for 1 bird (Tumulte), who was momentarily housed alone, subjects were housed together with their mate in outdoor aviaries of the Avian Cognition Research of the University of Oxford, hosted by the Max-Planck-Institute for Ornithology, Seewiesen, Germany (see Supplementary Information). Seven subjects had been wild-caught and had a minimum age of 5 years, and 2 were 1-year-old juveniles (Calypso and Crusoe). This study was approved by the ethics committee of the University of Lyon 2.

### Apparatus and Experimental Set up

For each experimental condition (Tool/Body) a test box was used in the experiment that consisted of a clear Perspex cube (10.8 × 15.2 × 6.5 cm) elevated on wooden planks (10.9 cm high) (Fig. 1). In the Body+/Tool− condition, the box had a hole of 3.1 cm in diameter at its back, located at 2.3 cm from its left side, through which the reward could be accessed directly with the hand/beak. The box front had a horizontal slot of 13.5 cm length into which the tool could be inserted in order to slide the reward along a horizontal rail track that had an opening at its end. The tool was presented on a wooden support (4 × 11.5 × 8.2 cm) at a predetermined distance from the box (crows: 60 cm, children: 1 m, human adults: 1m50). In the Body−/Tool+ condition, the reward could only be accessed with the hand/beak if two small windows were opened successively. The first window (5 cm high; 5 cm length) was larger and bigger than the second one (3.2 cm high; 2.9 cm length). For the crows, favored giant mealworms (*Zophobas morio*) were used as well-established motivating rewards^41,61,63–66^, which did not form part of the crows’ regular food. A little duck figurine (a highly attractive toy previously used in developmental studies on infants below two years of age^11^ but that appeared similarly motivating in older children) and a wrapped fifty euro-note were used as rewards for children and adults, respectively.

### Procedure

#### Habituation phase

*Crows*: Prior to the experiment, subjects were habituated to the apparatuses so as to reduce any neophobia as a potential confounding factor. The unbaited boxes and the platform were presented in the indoor enclosures near the food plates for 3 hours continuously.

#### Children

The apparatuses were placed in the classroom the day before the experiment, so the children were familiarized with the equipment before testing.

#### Pre-experience phase

Here, the subjects directly experienced the time and effort costs associated with both options (i.e. the hand/beak and the tool) of each condition (Body+/Tool− and Body−/Tool+), by exposing them to 2 versions of the 2 subsequent test boxes (4 versions in total; Figure S1). From one of these versions of each test box the food could only be obtained by using the hand/beak, whereas from the other version the food could only be obtained by using a tool. The order of the condition was counterbalanced (group 1 started with the Body+/Tool− condition first, whereas group 2 began with the Body−/Tool+ condition).

*Crows*: All phases took place in the indoor enclosures, which were cleared of all potential tools before each trial. Subjects learned how to retrieve the reward progressively, by trial-and-error learning. In order to pass criterion and move on to the test phase, subjects had to retrieve the reward 8 times in a row (trials of 12 minutes each) from each of the 4 versions, i.e. from the 2 versions of each condition.

*Human*: In this phase, upon entering the room, the subjects were instructed: ‘You stand still here’ (facing the task). The experimenter demonstrated how to get the reward using the Body and the Tool twice respectively, in each condition and in random order. The subject was then asked to get the reward twice with the Body and twice with the Tool in random order. With children, the experimenter attracted the subject’s attention by saying in an adapted tone of voice: ‘look into the box there is a little duck. But the little duck is stuck. To retrieve it, I can use my hand (demonstration × 2) or the tool (demonstration × 2) (in pseudo-random order). Once the task demonstration was complete, the participant was told: ‘now can you show me how did I retrieve the duck with the hand (experience × 2)? And with the tool (experience × 2)?’

#### Test Phase

For both of the actual test boxes used in the test phase, the features of the 2 versions of each condition previously used for training in the pre-experience phase were combined (see Supplementary Information). Hence, in contrast to the pre-experience phase, the subjects could now choose freely between either using the hand/beak or the tool. The presentation of the box varied pseudo-randomly.

*Crows*: All the subjects were tested individually in 24 trials of 8 minutes (12 trials per condition). Typically, 2 trials were conducted per day.

*Humans*: Testing took place in a calm and isolated room and the apparatus was set up in the centre of a table. Before each trial, subjects were instructed to stand still, facing the task with their arms and hands hanging down. A trial began once the subject raised a hand to solve the task. The subjects were given little breaks of a few minutes between trials, to ensure they continued to be motivated and kept attending to the task. Twenty test trials were realized per subject (i.e. resulting in 10 trials per condition). Each test lasted 5 minutes max. and ended when the subject reached the reward.

#### Additional tests for the New Caledonian crows

*Transfer task*: The apparatus was a modified replication of the multi-access box used by Auersperg *et al*. (2011)^63^. The reward was located on a pillar in the center of a wooden box, which was opened on the front side, and subjects could either take it directly with the beak or with a tool. Three trials for each subject were conducted and did not exceed 8 minutes each. It was assessed whether the subject used the tool or the beak first.

*Novel object exploration task*: The object, i.e. a soft toy bee, to which no subject had been confronted previously, was placed on the ground in the centre of the room. All objects except sticks were removed. Four sticks were added around the object. The test finished as soon as the crow touched the object with its beak or with a tool.

## Electronic supplementary material

Supplementary Information

Video 1. Body-/Tool+ - Tool option

Video 2. Body-/Tool+ - Body option

Video 3. Body+/Tool- - Body option

Video 4. Body+/Tool- - Tool option

Video 5. Exploration task - Body option

Video 6. Exploration task - Tool option

Video 7. Transfer task - Body option

Video 8. Transfer task - Tool option
