# Supplementary material for: From the Age of 5 Humans Decide Economically, Whereas Crows Exhibit Individual Preferences
Source: Sci Rep. 2017 Dec 6;7:17043. doi: 10.1038/s41598-017-16984-0 (PMC5719055; doi:10.1038/s41598-017-16984-0)
Supplement: Supplementary file 1 — Supplementary Information [file 41598_2017_16984_MOESM1_ESM.pdf]

Supplementary Information

From the Age of 5 Humans Decide Economically, Whereas Crows Exhibit Individual Preferences

Samara Danel, François Osiurak, and Auguste M.P. von Bayern

Methods

Subjects and Housing conditions

- Crows

Throughout the day, the subjects had *ad libitum* access to fresh water and food which consisted of a variable food mixture composed of minced beef heart, rice, oats, egg, insect mixtures, curd, vegetable oils, vitamins and minerals, as well as soaked cat biscuits, Versele Laga NutriBird® beo-pearls and fresh fruit. Crows were kept in outdoor aviaries (~25 m² each) with associated heated and lit indoor enclosures (~5 m² each). The indoor chambers were kept under a 12h: 12h light regime with Arcadia ® Bird lamps providing the UV spectrum of natural sunlight (see Table S1 for information about the subjects).

- Children

Eight 4-year-olds and 22 5-year-olds were tested at a preschool in Saint-Zacharie, France (N = 30; preschool 1). Nineteen 3-year-olds and one 4-year-old child were tested at a preschool in Villars-les-Dombes, France (N = 20; preschool 2). The experiments were carried out from the 15th of September to the 6th of November 2014 in preschool 1 (Saint Zacharie, France), and from the 1st of March to the 26th of March 2015 in preschool 2 (Villars-les-Dombes, France) (see Table S2 for information about the subjects).

- Adult humans

The experiments were carried out from the 8th to the 17th of March 2015 (see Table S3 for information about the subjects).

Materials

Pre-experience phase. The two versions of each test box (condition A: Body+/Tool: versions 1 & 2; condition B: Body-/Tool+: versions 3 & 4) consisted of a clear Perspex box (10.8 x 15.2 x 6.5 cm) elevated on wooden poles (10.9 cm high) (Figure S1).

Fig. S1.

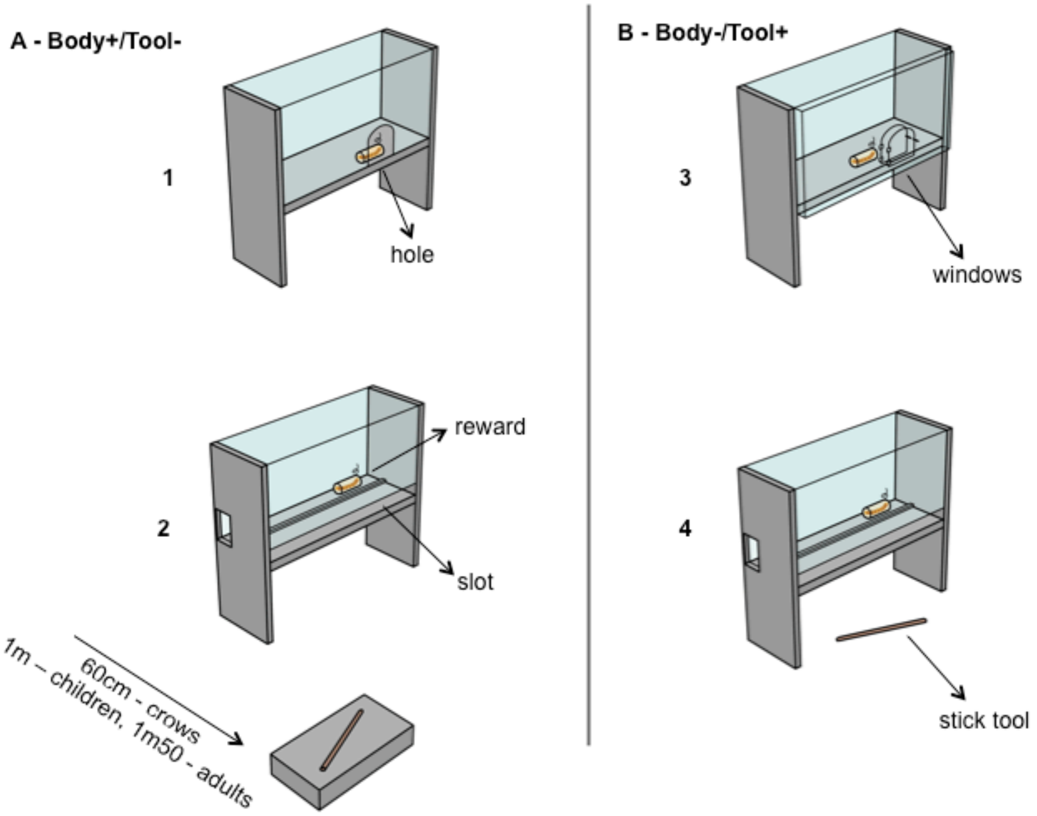

Fig. S1. Schematic representation of the 4 versions of the subsequent test boxes of each condition used in the pre-experience phase with crows. Drawing courtesy of Nicolas Brachet.

Panel A - depicts the Body+/Tool- condition. From the version 1, the reward could only be taken with the hand/beak directly through a hole of 3.1 cm in diameter, which was located at 2.3 cm from the right side of the box. From the version 2, the reward could only be retrieved with the tool i.e., a short stick. It had a horizontal slot along the entire length of the box, into which the tool could be inserted in order to slide the reward along a horizontal rail track at the backside of the box. The rail track had an opening at its end, through which the reward could fall out of the box. The tool was

provided on a wooden platform (4 x 11.5 x 8.2cm) away from the box (i.e. crows: 60cm, children: 1m, adult humans: 1m50).

Panel B - represents the Body-/Tool+ condition. From the version 3, the reward could only be retrieved with the hand/beak. It had two small windows, the first one (5 cm high; 5 cm length) was bigger than the second one (3.2 cm high; 2.9 cm length), so they had to be opened consecutively. The version 4 was identical to version 2, except that the short stick was placed in front of the box rather than at a distance.

Additional tests - the transfer task

The apparatus (i.e. the transfer box) consisted of a wooden box (22 cm high; 12.5 cm length) containing a pillar on which the reward was presented (Figure S2). The pillar's top could be accessed via a big hole located on the box's front side (6 cm high; 3 cm wide). The subject could either take the reward directly with the beak, or push it down out of the box by inserting a tool into the box. The box was placed on the ground in the middle of the room on a wooden platform (29cm x 14.5cm). Usually 4 to 7 sticks (from 8 to 15 cm of length; 2 mm in diameter) were placed around the box.

Fig. S2.

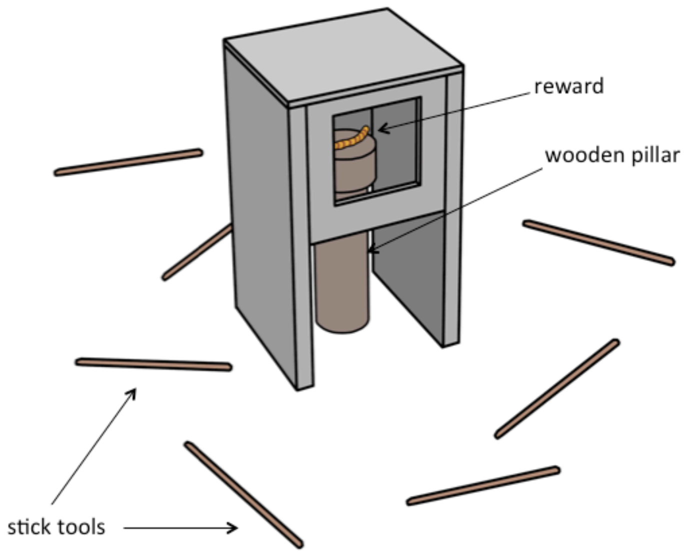

Figure S2. Illustration of the transfer box used during the transfer task of the additional tests. The subjects could either take the reward out of the hole located on

the front’s box directly with the beak, or take a stick from the ground and use it to push the reward on the floor. Drawing courtesy of Nicolas Brachet.

Experimental Setup and Procedures

- Pre-experience phase

Crows

Prior to testing in each condition, subjects gained pre-experience through exposure to 2 different versions of each subsequent test box (4 versions in total). This ensured that they knew about the time and effort associated with each option, before being allowed to choose between them in the subsequent tests. Hence, each version of the box could only be solved by a single technique (either tool or beak). In the Body+/Tool- condition, the effort to get the reward from the apparatus in the version 1 with the beak was lower than the effort involved in using the tool, which was placed away from the apparatus in the version 2. In the Body-/Tool+ condition in contrast, the effort to get the reward from the version 3 with the beak was greater because it involved the opening of two mutually blocking windows, compared to the version 4 in which the tool was presented just in front of the apparatus. Once the crows had retrieved the food from each version in their respective first condition 8 times consecutively, they moved on to the test phase in which the properties of both versions were combined into one test box for each condition (i.e., versions 1 & 2 = Body+/Tool-’s box; versions 3 & 4 = Body-/Tool+’s box).

- Additional tests for the New Caledonian crows

Novel object exploration task

An object with a smooth, continuous surface was chosen so as to reduce the probability that the crows would interact with the object in an attempt to extract food.

Data Analysis

Except for the subjects at the preschool 1, for which we did not have an authorization for filming, each trial was filmed with a Samsung HMX-F90 and a Panasonic NV-GS60, (all original films of crows, children from the preschool 2 and adult humans have been deposited into long-term storage within the Laboratory for the Study of Cognitive Mechanisms, University Lumière Lyon 2).

Results - pre-experience phase

As the data satisfied the conditions of normality, parametric statistical tests were conducted using paired *t*-tests with 3- to 5-year-olds and adult humans, in order to assess if subjects took less time to reach the reward with the more efficient option in each condition. The significance level was set at $p < .05$ (Figure S3). In the Body+/Tool- condition, we found that using the hand/beak was faster than using the tool (crows, Wilcoxon paired *T*-test: $Z = 0$, $p < .02$; 3-year-olds, Student *t*-test: $t(19) = 13.59$, $p < .001$; 4-year-olds, Student *t*-test: $t(9) = 18.37$, $p < .001$; 5-year-olds, Student *t*-test: $t(22) = 20.83$, $p < .001$; adults, Student *t*-test: $t(15) = 28.67$, $p < .001$). In the Body-/Tool+ condition, using the tool was faster than using the hand/beak (crows, Wilcoxon paired *T*-test: $Z = 2.52$, $p < .02$; 3-year-olds, Student *t*-test: $t(19) = 3.26$, $p < .01$; 4-year-olds, Student *t*-test: $t(9) = 4.48$, $p < .001$; 5-year-olds, Student *t*-test: $t(22) = 20.83$, $p < .001$; adults, Student *t*-test: $t(15) = 3.69$, $p < .01$).

Fig. S3.

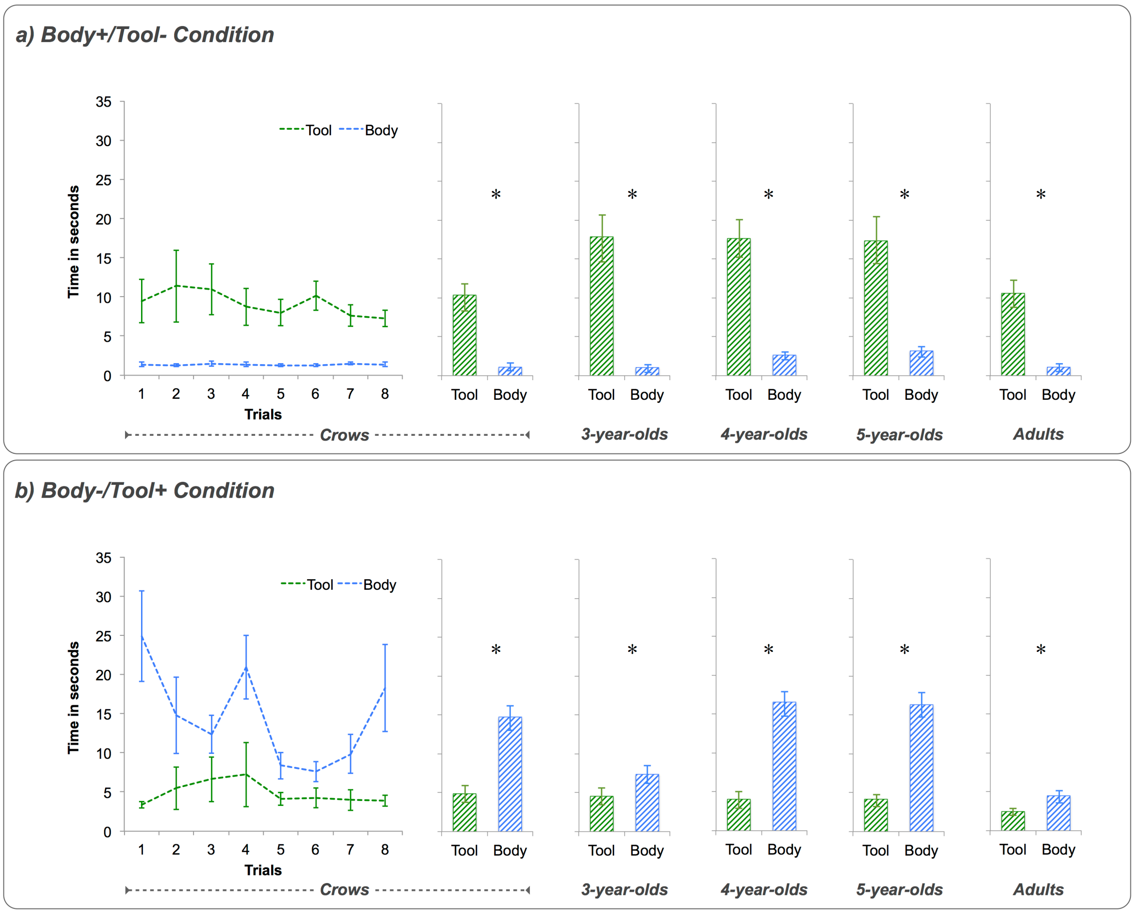

Fig. S3. Time spent reaching for the reward with the hand/beak versus the tool in both conditions during the pre-experience phase for crows, children and human adults. The bars represent the standard error deviation.

Table S1. Information about the New Caledonian crows.

Age is reported in years. Group 1 was tested in the Body-/Tool+ condition first, and then moved to the Body+/Tool- condition, group 2 was tested in the opposite order.

Subjects	Name	Age	Sex	Group
1	Jungle	≥ 5	Male	2
2	Liane	≥ 5	Female	2
3	Tortue	≥ 5	Female	2
4	Crusoe	1	Male	2
5	Papaye	≥ 5	Male	1
6	Tabou	≥ 5	Female	1
7	Tumulte	≥ 5	Female	1
8	Calypso	1	Female	1

Table S2. Information about the participating children.

***a)** Information about the 4- and 5-year-olds in preschool 1.* The group number denotes in which order the conditions were presented to each subject (1 = Body-/Tool+ first; 2 = Body+/Tool- first).

Subjects	Age (years)	Sex	Group
1	4	Male	1
2	4	Female	2
3	4	Female	1
4	4	Female	2
5	4	Male	1
6	4	Female	2
7	4	Male	1
8	4	Male	2
9	5	Female	1
10	5	Female	2
11	5	Male	1
12	5	Female	2
13	5	Male	1
14	5	Female	2
15	5	Female	1
16	5	Male	2
17	5	Male	1
18	5	Male	2
19	5	Female	1
20	5	Female	2
21	5	Female	1
22	5	Male	2
23	5	Female	1
24	5	Female	2
25	5	Female	1
26	5	Male	2
27	5	Female	1
28	5	Female	2
29	5	Female	1
30	5	Female	2

b) *Information about the 3- and 4-year-olds in preschool 2.* The group number denotes in which order the conditions were presented to each subject (1 = Body-/Tool+ first; 2 = Body+/Tool- first).

Subjects	Age (years)	Sex	Group
1	3	Female	1
2	3	Female	2
3	3	Male	2
4	3	Female	1
5	3	Female	2
6	3	Male	2
7	3	Male	1
8	3	Female	1
9	3	Female	2
10	3	Male	1
11	3	Female	2
12	3	Male	1
13	3	Male	1
14	3	Female	2
15	3	Female	2
16	3	Male	1
17	3	Female	1
18	3	Male	2
19	3	Male	1
20	4	Female	2

Table S3. Information about the adult humans.

The group number denotes in which order the conditions were presented to each subject (1 = Body-/Tool+ first; 2 = Body+/Tool- first).

Subjects	Age (years)	Sex	Group
1	21	Female	1
2	22	Male	1
3	25	Female	2
4	27	Female	1
5	27	Male	2
6	28	Female	2
7	29	Female	2
8	29	Female	2
9	32	Female	1
10	35	Male	2
11	38	Male	2
12	40	Female	1
13	44	Male	1
14	45	Female	1
15	57	Male	1
